# Noise Tolerant Photonic Bowtie Grating Environmental Sensor

**DOI:** 10.1021/acssensors.3c02419

**Published:** 2024-04-10

**Authors:** Kezheng Li, Nyasha J. Suliali, Pankaj K. Sahoo, Callum D. Silver, Mehmet Davrandi, Kevin Wright, Christopher Reardon, Steven D. Johnson, Thomas F. Krauss

**Affiliations:** † School of Physics, Engineering and Technology, University of York, Heslington, York YO10 5DD, U.K.; ‡ Reading Technical Centre, Procter and Gamble Technical Centres Ltd., Reading RG2 0QE, U.K.; § Department of Physics, Dhenkanal Autonomous College, Dhenkanal 759001 Odisha, India

**Keywords:** optical sensor, guided-mode resonance, bowtie grating, temperature compensation, mechanically robust sensor

## Abstract

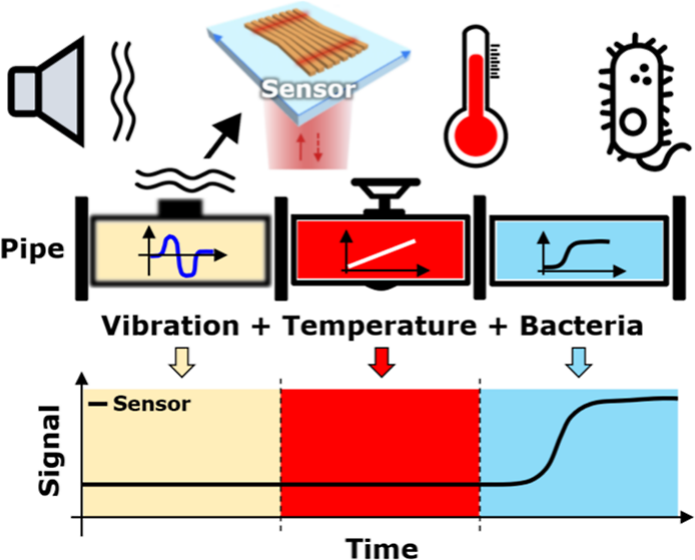

Resonant photonic refractive index sensors have made major advances based on their high sensitivity and contact-less readout capability, which is advantageous in many areas of science and technology. A major issue for the technological implementation of such sensors is their response to external influences, such as vibrations and temperature variations; the more sensitive a sensor, the more susceptible it also becomes to external influences. Here, we introduce a novel bowtie-shaped sensor that is highly responsive to refractive index variations while compensating for temperature changes and mechanical (linear and angular) vibrations. We exemplify its capability by demonstrating the detection of salinity to a precision of 0.1%, corresponding to 2.3 × 10^–4^ refractive index units in the presence of temperature fluctuations and mechanical vibrations. As a second exemplar, we detected bacteria growth in a pilot industrial environment. Our results demonstrate that it is possible to translate high sensitivity resonant photonic refractive index sensors into real-world environments.

Photonic refractive index sensors are well established as a choice technology for the instantaneous, label-free, contactless, and highly sensitive characterization of many technologically relevant analytes. There is high demand for such sensors in the biological, chemical, environmental, defense, transport, and food industries, both for the detection of specific targets and also in the area of process control.^1−3^ Refractive index sensors typically employ the overlap of the evanescent tail of a guided mode with the analyte to detect small changes, either with or without a binder molecule attached to the surface for increased specificity.^4,5^ The implementation of such sensors in a real industrial environment is limited, however, by their sensitivity to other external influences such as mechanical vibrations and temperature variations; these external influences generate significant noise, which may easily screen the desired signal(s). For example, mechanical vibrations often limit the use of sensors to vibration-free environments,^6−12^ which are impractical for applications in the field such as in industrial plants. Some sensor architectures such as photonic crystal fiber grating refractive index sensors^13^ achieve very high refractive index sensitivity, in the 1 × 10^–6^ range, and mechanical tolerance, but they are not temperature compensated and require the analyte to be infiltrated into the fiber as well as a high performance spectrometer for the readout, which limits these sensors to laboratory research. The susceptibility of photonic sensors to thermal fluctuations is also well-known,^14−17^ especially in silicon-based sensors due to silicon’s high thermo-optic effect of d*n*/d*T* = 1.8 × 10^–4^ 1/K.^18−22^ A common solution to the temperature dependence of silicon photonics is to use a polymer coating that exhibits the opposite thermo-optic effect, thereby making the device largely temperature insensitive.^23,24^ Such polymers, however, are usually not compatible with some of the harsher biological or industrial environments where the sensor needs to operate; moreover, the coating may compromise the operation of the sensor by screening the evanescent tail of the mode. Other researchers have incorporated structure compensation,^25,26^ measured fringe contrast^27,28^ and implemented dual micro resonators^29,30^ in Fabry–Perot fiber sensors to compensate for temperature variations, achieving limits of detection in the range of 10^–5^ to 10^–3^ refractive index units. None of these sensors, however, are able to compensate for both mechanical and thermal vibrations, which limits their use to vibration-free platforms, and they are therefore incompatible with industrial plants. Our modality is unique in achieving both temperature and mechanical noise compensation while being portable. The only sensor we are aware of that compensates for both mechanical vibration and temperature is the critical angle refractometer described by Guo et al.,^31^ which operates by introducing a reference glass of known refractive index. This approach is laboratory-based and achieves a limit of detection (LOD) of 2.5 × 10^–4^ refractive index units. The question is whether a sensor can be developed with a similar or better performance that can also be used in the field.

Here, we introduce a novel bowtie-shaped sensor, which offers very high sensitivity to target refractive index variations while being tolerant to both mechanical and thermal noise. The sensor exploits the well-known concept of guided-mode resonances (GMRs), together with the chirped modality first introduced by our group.^32^ We demonstrate the ruggedness of the sensor by measuring salinity and bacteria growth in the presence of thermal and mechanical noise. Our results demonstrate that it is possible to translate high sensitivity resonant photonic refractive index sensors into real-world environments. Our work offers a method for building cost-effective, high performance, and resilient environmental sensors that are suitable for monitoring the growth of bacterial biofilms in water, in industrial settings, or in the environmental domain. Such sensors may find applications in many domains, such as the manufacture of home and personal care products, food/drink, water testing, or the Pharma industry.

## Results and Discussion

### Design

**Figure 1:**
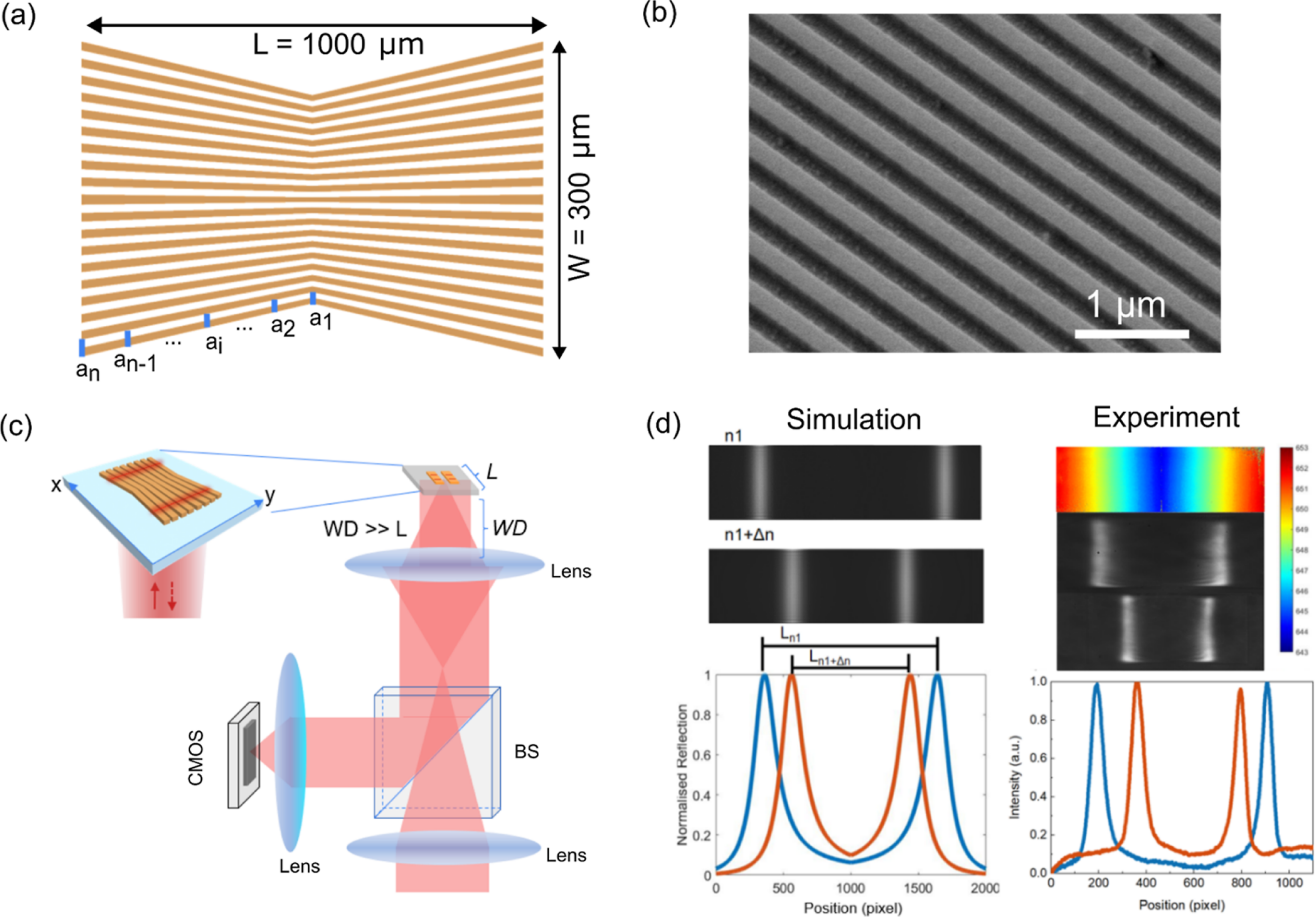
Illustration of the bowtie grating. (a) Top view of the bowtie chirped-GMR grating. The period *a* increases gradually from the center toward both edges with a step of 1 nm. For illustration purposes, the schematic graph is not to scale. (b) SEM image of a region of the bowtie GMR. (c) Bespoke inverted microscope for characterizing the bowtie chirped GMR. The microscope consists of an objective with a focal length of 16 mm, NA = 0.25, and a tube lens with a focal length of 150 mm. The distance between the objective and the tube lenses is 180 mm. Since the working distance of the objective lens (WD = 10.6 mm) is much longer than the size of the sensor (L = 1 mm), the lateral movement of the sample with respect to the optical axis is the strongest effect. (d) Simulated resonant image before and after the surrounding (refractive index) change. The left bottom curve shows the profile of the resonant image. The right panel consists of the measured hyperspectral image (top) and the experimental resonance image (middle and bottom) with resonance profile.

The bowtie grating consists of two opposing chiral GMR structures. We vary the period continuously in the opposite *x*-directions, starting from the center line, as shown in Figure 1. The concept of chirping the GMR grating is to convert spectral information into spatial information, allowing for easy readout of the resonance position with a CMOS camera. The sensor is illuminated by a collimated beam, and the reflected signal is collected by a beam splitter. The sensor has a smaller period (*a*_1_) at the center and the period gradually increases toward the outer regions (*a*_*n*_). When the refractive index of the analyte changes, the position of the resonance moves laterally to satisfy the resonance condition λ_res_ = *n*_eff_*a*, where λ_res_ is the resonance wavelength, *a* the local period, and *n*_eff_ the effective index of the guided mode, *n*_eff_ being a function of the overlap integral of the mode with the analyte. By measurement of the distance between the two opposing bars (Figure 1b), the refractive index of the analyte can be determined accurately. Due to the unique mirror symmetry, the bowtie grating structure can effectively cancel out mechanical noise, while doubling the spatial sensitivity; mechanical movement translates both bars in the same direction, while refractive index increases the distance between the bars. In Figure 1a, a segment of the bowtie grating is illustrated, showing a period range from 426 to 434 nm and a filling factor of 70%. The sensitivity was determined by measuring different concentrations of an ethanol solution, as shown in Figure S1.

### Vibration Noise Reduction

**Figure 2:**
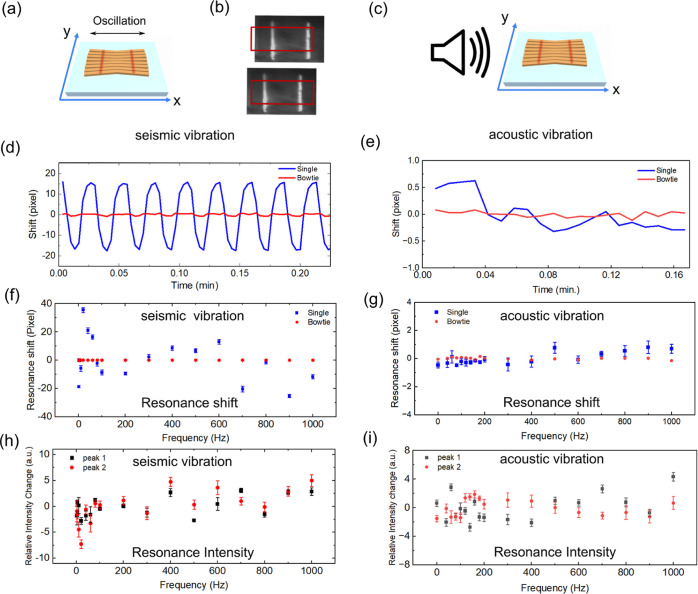
Seismic and acoustic vibration test for a bowtie GMR sensor. Here, seismic vibration refers to the mechanical vibration directly applied to the sensor along the *x* axis. Acoustic vibration was generated by a loudspeaker placed against the sensor at around 5 cm away, which generates vibrations in all three spatial directions (for more detail, see Figure S10). The sketches illustrate (a) the vibration along the *x* axis for the bowtie GMR sensor. The sample was placed on the stage which applied a repeatable vibration along *x*, *y*, and *z* directions. (b) The resonant image drifts away from the ROI, which is indicated by the red rectangle. (c) Illustration of the acoustic vibration setup (for more detail, see Figure S10). The vibration is generated by a loudspeaker with an output power of 103 dB and a random noise generator. The speaker is placed at the edge of the sensor, the output acoustic wave is along the *x* axis. (d) The resonance shift as a function of time under (d) seismic vibration and (e) acoustic vibration. The blue curve represents a single chirped GMR and the red curve represents the bowtie grating. The vibration frequency is set to 40 Hz with an amplitude of 1 μm. (f) Dependence of resonance shift on seismic vibration frequency. The maximum travel range is set to 20 μm. (g) Dependence of resonance shift on acoustic vibration frequency. It shows that the bowtie sensor suppresses acoustic vibration much better than the single GMR sensor. The standard deviation for the resonance shift of a single GMR and a bowtie GMR are 0.32 and 0.05 px, respectively, which represents a 6-fold improvement. The maximum acoustic output power is 103 dB. (h) Dependence of relative resonance intensity on seismic vibration frequency. (i) Dependence of relative resonance intensity on acoustic vibration frequency. Note, the relative resonance intensity is calculated as the disparity between the actual resonance intensity and the average value for each data set. This approach facilitates a clearer visualization of error bars. A total of 25 repeat measurements were conducted at each frequency to ensure robust and comprehensive data collection.

In general, vibrational noise includes seismic (ground) vibrations, acoustic vibrations (generated by air compressors, pumps, fans, etc.), and forces exerted on the sensor/sample. However, for the purpose of our analysis, we concentrate specifically on seismic and acoustic noise cancellation as these factors serve as the main influences that could affect the performance of the sensor in our design. We note that in all cases the bowtie sensor is mechanically fixed into the optical path. Nevertheless, because any lens needs free space to form an image and requires space for an illumination-path, the sensor cannot be hard-mounted to the camera. This is representative of many industrial applications, which only allow for a single access point through a single window, so we cannot operate with transmissive illumination, as e.g., many “lens-less” systems^33^ do. Therefore, we must operate in reflection, which makes the setup subject to vibrations. These vibrations for a simple chirped sensor are clearly shown in Figure 2, together with their mitigation by the bowtie configuration.

To investigate the impact of seismic (mechanical) movement, we placed the sensor onto a motorized linear translation stage and moved it along the *x*, *y*, and *z* axes with different frequencies and travel ranges, as indicated in Figure 2a. Figure 2b displays the resonance images obtained during the oscillation. Notably, the positions of the white bars have shifted, relocating to various positions within the region of interest (ROI). In Figure 2d, the resonance position is depicted over time for both the single chirp GMR and the bowtie GMR. Specifically, the vibration frequency is configured at 40 Hz, with a maximum travel range of 1 μm. Given the bowtie GMR geometry (depicted in Figure 2a), the predominant impact of mechanical vibrations is expected to be a lateral displacement of the sample in the *x*-direction. This is because the optical axis (*z*-direction) significantly exceeds the size of the sample, as illustrated in Figure 1a. Additionally, movement along the *y*-direction does not influence the readout, since the distance between the resonance bars is measured in the *x*-direction. Consequently, the *x*-direction is the most vulnerable to vibrations, which is the focus of our presentation here. The movement along other directions are shown in the Supporting Information (Figures S2–S9). Figure 2d shows that the bowtie configuration reduces mechanical noise from 15 pixels to less than 1 pixel, which illustrates the fact that the distance between the two resonant bars does not change when the sensor moves, while their absolute position does. Consideration of the vibration frequency dependence is crucial. In Figure 2e, a comparison of resonance shifts across various seismic vibration frequencies (1–1000 Hz) with a 20 μm maximum travel range is presented. It is evident that the bowtie GMR sensor maintains a consistent resonance shift across frequencies, whereas the single chirp GMR is more significantly affected. Additionally, our exploration of angular misalignment effects (Section 1.3 of the Supporting Information) reveals that the bowtie configuration serves as a mitigation strategy. Figure 2e illustrates the impact of acoustic vibrations on the sensor at different frequencies. While acoustic vibration has a lesser effect on the sensor’s resonance shift for both single and bowtie GMR, the bowtie GMR’s error bar is 5.7 times smaller than that of the single GMR. Furthermore, the resonance shift in the bowtie GMR remains more constant than in the single GMR, indicating that bowtie GMR’s could reduce acoustic noise more efficiently. It is noted that the amplitude of the bowtie sensor remains relatively consistent across varying vibration frequencies, as depicted in Figure 2h,i for both seismic and acoustic vibration.

### Temperature Compensation

**Figure 3:**
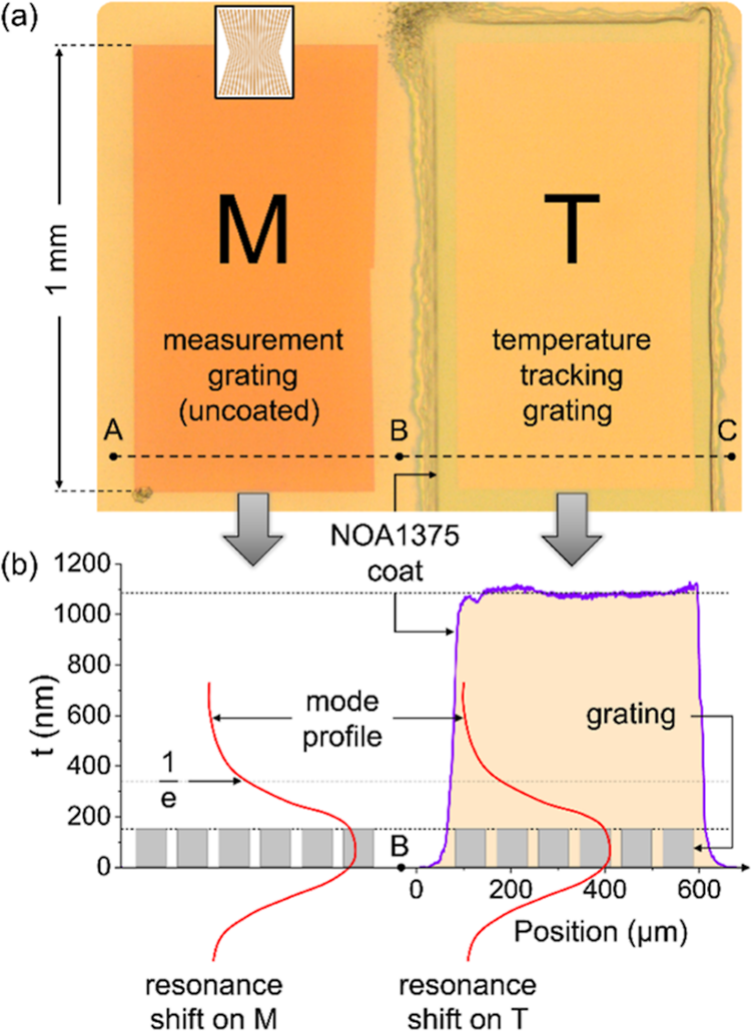
(a) Optical micrograph of the sensor showing the uncoated grating M in contact with the analyte as well as the coated grating T. The inset indicates the orientation of the chirp. (b) Line profile taken across points B and C to show the average thickness (*t*) of the coating of 1090 nm. We also indicate the evanescent tail of the resonant mode, which is able to interact with the analyte in regions A-B but only with the polymer coating in regions B-C (Figure S11 shows that the 1/*e* decay length of the mode is approximately 180 nm). Typical resonance images are shown in Figure 4.

The second intervention we introduce compensates for temperature changes. The principle is to cover a reference grating with a sufficiently thick material such that the evanescent tail does not see the analyte. The response of this reference grating is then only dependent on the thermo-optic coefficient of the coating and not on the analyte. Figure 3a shows an optical micrograph of the arrangement where grating T is the reference grating, while grating M is in contact with the analyte, so its response depends on both temperature- and analyte composition-dependent refractive index changes. We refer to the temperature-dependent index change of the reference grating as Δ*n*_T_, the index change measured from the analyte as Δ*n*_M_, from which the desired, temperature-compensated index Δ*n*_C_ is determined. We coat grating T with Norland NOA1375 because Norland UV-curable glues can be selected at various refractive indices and are highly durable when cured; we use a thickness of approximately 1 μm to ensure that the evanescent tail of the GMR no longer sees the analyte. Details of the coating process are in Section 2.1 of the Supporting Information. Figure 3b shows the thickness of the coating via a line profile obtained by surface profilometry, together with a simplified mode profile. Since the thermo-optic coefficient for the coating is different from that of the analyte, sensors need to be calibrated first, but once this is done, Δ*n*_C_ can be determined accurately, as we show next.

**Figure 4:**
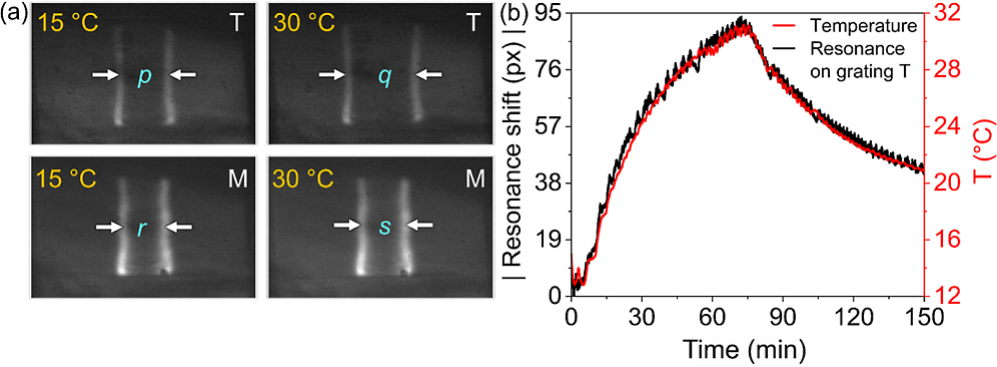
(a) Resonance images of gratings T and M at 15 °C and 30 °C. The average separation of the resonance peaks are *p* = 414 px, *q* = 582 px, *r* = 352 px, and *s* = 374 px. The absolute resonance shift for the 15 °C rise is 84 px on T and 11 px on M. (b) Plots of the time-varying temperature (*T*) and magnitude of resonance shifts at grating T. The temperature compensation factor, which accounts for the difference in TOE coefficients is discussed in Section S2.2.

**Figure 5:**
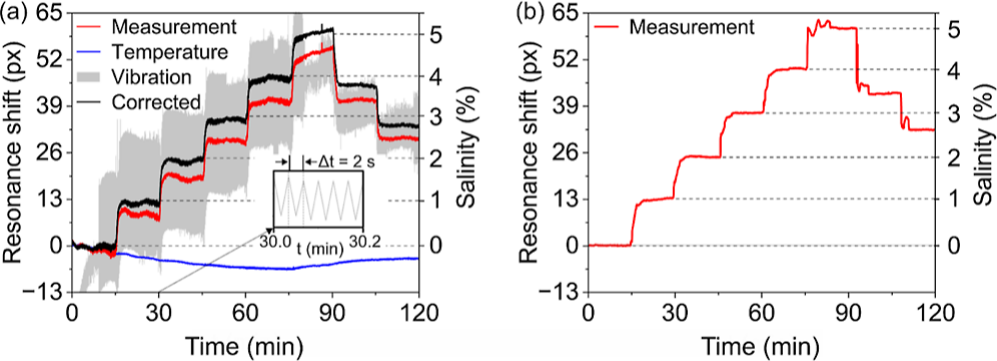
(a) Measurements of salinity (red curve) increased in steps of 1% while temperature (blue curve) varies from 15 to 30 °C and the sensor vibrated at 0.5 Hz (gray curve) along the grating (axis joining the pixel distance arrows in Figure 4a). The inset shows 6 cycles of the raw resonance oscillations on M (gray graph), from which the period of oscillations can be found. The bowtie sensor reduces the peak-to-peak vibrational noise by 97.3% (31 dB) without applying any digital filtering functions. (b) Resonance measurements (red curve) obtained as the salinity is increased in steps of 1% in the absence of temperature variations and vibrations. Note the close agreement with the compensated (black) curve in panel (a).

Figures 4a,b and 5 demonstrate the successful operation of the temperature compensation method (see experimental details in Section S2.2 and setup in Figure S13). We first show exemplar resonance images obtained from the bowtie gratings to illustrate the resonance shift due to temperature at the start and at the end of the heating cycle with the analyte (here: water) unchanged. The gap between the resonance bars on both the T and M gratings increases, but the increase on the M-grating is smaller than on T. This difference is due to the fact that the thermo-optical coefficient of water is smaller than that of the NOA1375 polymer. The calibration procedure to account for this difference is outlined in the Supporting Information, Section S2.2 and illustrated in Figure S14. In Figure 4b, we then plot the normalized resonance shift as a function of temperature (black curve) and compare it to the controlled water temperature (red curve). The two plots are clearly in phase, as expected; we analyze the linearity in Figure S15. Note that both curves appear noisy, which is due to the temperature control feedback circuit, which turns the heater on and off to achieve the desired heating curve; the position of the resonance curve accurately tracks this oscillation. We show further proof of the stability over longer periods in Figure S16.

### Salinity Measurement in the Presence of Vibrations and Temperature Variations

We now demonstrate the utility of the compensation methods described above to the salinity measurement. Salinity measurements are important in many areas of environmental and marine science such as global warming,^34−36^ ecological variations,^37−39^ and biotic activities.^40−42^ The refractive index of seawater changes by Δ*n* ≈ 2 × 10^–3^/% salinity,^43^ so the demonstrated sensitivity of a GMR sensor in the low 10^–5^ range^4^ allows the determination of salinity with high accuracy. Salinity measurements are typically conducted in the field and involve mechanical vibrations and temperature fluctuations; hence, salinity measurements are ideally suited to showcasing the benefit of the compensation methods introduced here.

Figure 5 summarizes the result. We exposed the sensor to water of different salinity (changed every 15 min in steps of 1%) while vibrating it at 0.5 Hz and heating it up from 15 to 30 °C. In Figure 5a, the raw data shows the strong noise in the resonance, introduced by the mechanical vibration, while the blue curve shows the additional resonance shift due to temperature. The bowtie geometry allows us to remove both, first the mechanical vibration (red curve), then temperature, resulting in fully compensated data (black curve).

In these noisy conditions and once compensation has been applied, the sensor’s bulk sensitivity is 98.9 nm/RIU or 0.19 nm/% salinity (see results in Figures S17 and S18) which is in agreement with recently reported photonic salinity sensor sensitivities in the range of 0.06–5 nm/%.^44−47^ Its LOD is then 0.1% salinity or 2.3 × 10^–4^ RIU. For reference, Figure 5b shows measurements obtained while the sensor was neither heated nor vibrated. In this control experiment, the LOD = 0.03% salinity or 6.5 × 10^–5^ RIU which is consistent with previous measurements with the chirped GMR.^32^ In the presence of noise, the LOD increases to 2.3 × 10^–4^ RIU, a 3-fold increase, which is impressive considering the large disturbances to which we exposed the sensor to. This LOD translates into a measurement of salinity to a precision of 0.1% making the sensor competitive for monitoring desalination plants and an important contribution to salinometer precision which is still to be standardized.^48^

### Temperature-Compensated Bacteria Growth

**Figure 6:**
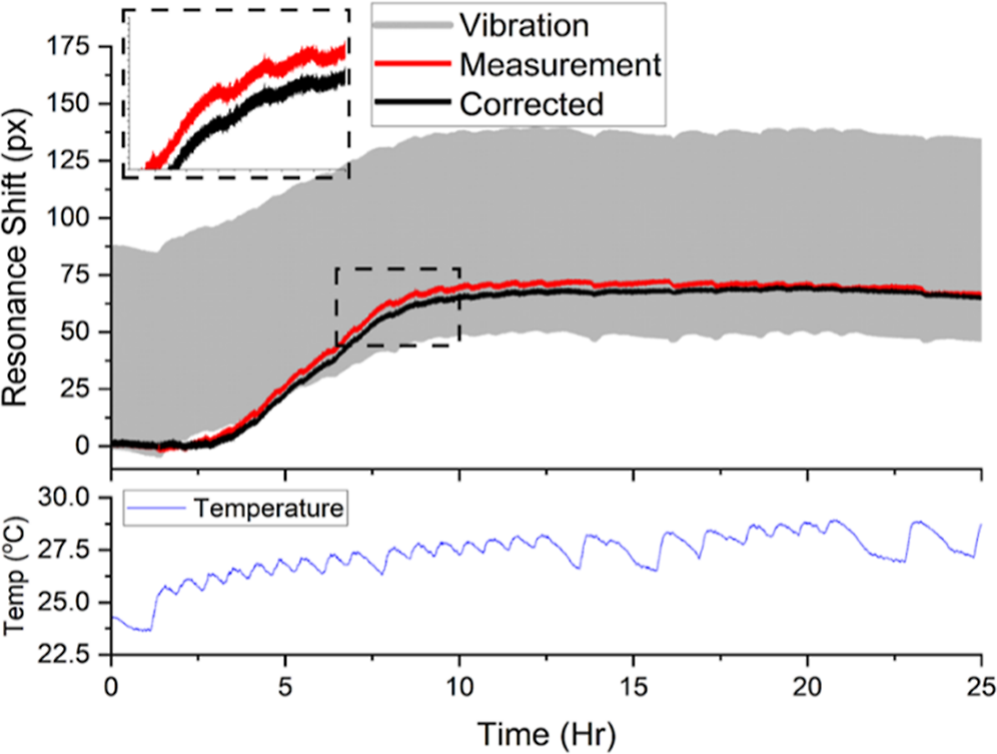
Measurement of *S. aureus* growth over 25 h using the temperature-compensated bow-tie sensor. Gray—output measured from one resonance of the bowtie sensor illustrating a typical chirped-GMR output measurement; red—output measured by taking the separation of the bowtie sensor resonances; black—output from the bowtie sensor compensated for temperature effects using the NOA coated bowtie. The temperature graph (blue) shows the temperature near the sensor resulting from the on/off cycles of the heater. The zoom-in exemplifies this temperature-related fluctuation.

Having now demonstrated the capability of the system, we challenged the sensor by measuring the growth of *Staphylococcus aureus* in a mechanically and thermally noisy environment. A solution containing approximately 10^8^ cfu/mL *S. aureus* was prepared using a BioBall MultiShot 10 × 10^8^ (Biomérieux), as described in Section S2.3. 50 μL of the BioBall solution was injected into a sterile microwell (Ibidi—80366) affixed to the sensor containing 30 μL PBS and 20 μL TSB (30 g/L). To simulate the noisy environment, the stage of the measurement instrument was programmed to oscillate with a 100 μm amplitude at a frequency of 0.2 Hz in the *x*-direction and the sensor and analyte were heated periodically using an electric heater. The sensor afforded the removal of almost all of the environmental noise, reducing the standard deviation of a typical chirped-GMR signal from 42.9 to 0.94 px, revealing a clear growth curve over 25 h. Figure 6 shows the response. The measurement of half of the bowtie sensor (Figure 6 gray) illustrates the output of a typical chirped-GMR sensor, clearly highlighting the benefit of the bowtie sensor in mechanically noisy environments. Temperature fluctuations of the sensor are illustrated by Figure 6—red, where the signal shows oscillating trends that match the oscillation of the temperature sensor (Figure 6—blue). Without compensation, these oscillations could be misinterpreted for bacterial growth changes; however, after compensation, these oscillations are significantly reduced (Figure 6—Black).

Once the temperature and mechanical shifts are accounted for, we can see a clear bacterial growth curve. It is well understood that bacteria exhibit a lag-phase before log-growth occurs; hence, the delay of a few hours before a change in signal is registered. Once the bacteria begin to grow, after 2–3 h, we observe a clear shift in the resonance. Eventually, the resonance saturates, and no further growth is detected. We expect this is a result of either a stationary growth phase, or the bacteria exceeding the sensing volume of our evanescent field sensor.^49^ This result demonstrates that the bowtie sensor is suitable for environmental and industrial applications, where the in-line monitoring of bacterial growth is a major issue, for which there is currently no solution.

**Figure 7:**
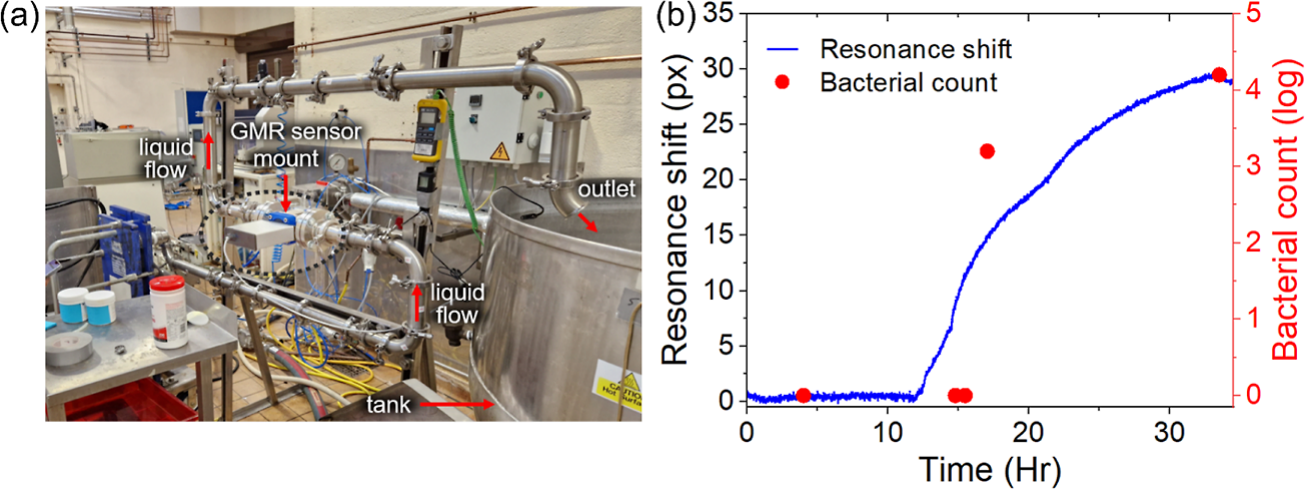
(a) A pilot industrial setup showing the bowtie grating sensor location on an industry standard pipeline. The sensor is mounted in-line on the pipe, which drives the process liquid to a storage tank at 93 L/min. (b) Biofilm measurements recorded on the sensor and on stainless steel coupons extracted in parallel. Note that the vertical axes are scaled to match at the onset and upon saturation, so the graph shows only qualitative agreement.

To further demonstrate the compatibility of our sensor with real-world environments, we demonstrate the monitoring of bacterial growth in a noisy environment, typical for industrial settings. As an example, we have installed a bowtie-grating sensor into a pilot industrial setting (Figure 7a), where mechanical vibration and temperature fluctuations are substantial. The setup comprises pipework with a section of pipe into which the sensor system is mounted. The flow rate of the process liquid is maintained at 93 L/min, and the pumps to drive this flow are part of the closed loop; the tubing noticeably vibrates. Figure 7b shows measurements obtained over 33 h. The process liquid is spiked with bacteria at a concentration of 1 × 10^6^ cfu/mL at time 0 and we observe the onset of a biofilm at *t* ≈ 12 h. In parallel, we extracted stainless steel coupons suspended in the liquid at regular intervals and determined the number of bacteria by observation under an optical microscope. The data show good agreement between the resonance shift and coupon count. The growth rate then saturates at approximately 30 h indicating that the growth is extending beyond the reach of the optical sensor, which only samples near-surface events.^49^ By comparing Figures 7b with 6, we note that the SNR of the measurement is comparable to that of the laboratory setup, indicating that we achieved successful noise mitigation in a real industrial environment.

Detection of bacterial contamination with a setup such as that shown in Figure 7 is crucial in many industrial settings. For example, most cases of bacteria-based infection in the USA have been attributed to food-borne bacteria^50−52^ making it imperative for food processing plants to quickly detect and mitigate against microbial activity. Further to this, regulations for preservative use in manufacturing and food-safety are regularly updated, shifting toward the use of nontoxic and environmentally sustainable preservatives.^53−55^ Given this background, there is a need for effective in-line monitoring to maintain hygienic manufacturing conditions and as an early warning of contamination to minimize the impact on production and reducing material waste. Current monitoring methods for detection are offline; therefore, developing an in-line measurement method is highly desirable.

## Conclusions

We have introduced a novel type of resonant biosensor, which we refer to as a bowtie grating. The bowtie grating exploits the phenomenon of guided-mode resonances, and we demonstrate its key feature, i.e., insensitivity to mechanical noise. When paired with another similar grating that is coated with a polymer, the combination offers additional temperature compensation. This arrangement allows us to conduct temperature- and vibration-insensitive measurements with high sensitivity. We demonstrate this capability by measuring salinity to high accuracy, i.e., with a LOD = 0.1% salinity (2.3 × 10^–4^ refractive index units) in the presence of a strong mechanical and thermal background, compared to LOD = 0.03% in a stable environment. When compared to vibration and temperature-compensating photonic sensors we cited, the bowtie sensor stands out as a cost-effective sensor that simultaneously compensates for both thermal and mechanical noise but retains high sensitivity. In addition, we demonstrate the growth of bacteria under conditions representing an industrial environment, including thermal and mechanical background, again able to extract the true growth curve. Clearly, this demonstrates the suitability of our sensor for industrial applications where thermal fluctuations and mechanical vibrations are omnipresent.

We note the importance of this work in the context of many other refractive index sensors, which have been published over the years. Many sensor modalities have been introduced, and they may show better performance than the performance we show here but are usually tested under laboratory conditions. We suggest that it is essential for the field of environmental sensors to move forward if it gains acceptance in a “real-world” environment. We hope that our work will make a contribution to this vision.

## Supporting Information Available

Section S1—fabrication and mechanical vibration analysis: S1.1, sensor materials, fabrication, and simulation; S1.2, vibration noise reduction using bowtie chirped GMR (Figure S1, sensitivity of bowtie chirped GMR sensor with different refractive indices corresponding to different concentrations of ethanol in water solution; Figure S2, mechanical vibration of GMR sensor along *y* axis; Figure S3, mechanical vibration along the *x* axis and an example of the resonance images; Figure S4, vibration along the *z* axis; Figure S5, comparison of the standard deviation of the resonance shift for bended bar chirped GMR; Figure S6, effect of ROI in the bended bar bowtie sensor; Figure S7, vibration along the diagonal of the x and *y* axis); S1.3, angle response for bowtie chirped GMR (Figure S8, illustration of rotation angle; Figure S9, angular tolerance test for bowtie chirped GMR; Figure S10, acoustic vibration setup). Section S2—temperature compensation: S2.1, fabrication of NOA1375 coatings and characterization; S2.2, temperature compensation setup and data processing; S2.3, bowtie and temperature-compensated bacteria growth. Other figures—Figure S11, cross section of the electric field distribution of a GMR; Figure S12, a typical resonance image of gratings T and M showing a typically low contrast resonance on T compared to M; Figure S13, position of the GMR sensor S and the temperature compensation setup; Figure S14, simulation of the temperature compensation signal processing technique; Figure S15, resonance shift plotted as a function of temperature; Figure S16, resonance response to temperature variation; Figure S17, resonance measurements of *V*_C_ obtained at 10 min intervals in the first hour and then at 15 min intervals; Figure S18, resonance measurements of temperature compensated resonance on M correlated to the RIU to verify sensitivity of the sensor (PDF)
(MP4)
