# Supplementary material for: Noise Tolerant Photonic Bowtie Grating Environmental Sensor
Source: ACS Sens. 2024 Apr 10;9(4):1857–65. doi: 10.1021/acssensors.3c02419 (PMC11059099; doi:10.1021/acssensors.3c02419)
Supplement: Supplementary file 1 — se3c02419_si_001.pdf [file se3c02419_si_001.pdf]

SUPPLEMENTARY INFORMATION

Noise tolerant photonic bowtie grating environmental sensor

Kezheng Li[a,*], Nyasha J. Suliali[a,*], Pankaj K. Sahoo[a,c], Callum D. Silver[a],

Mehmet Davrandi[b], Kevin Wright[b], Christopher Reardon[a], Steven D. Johnson[a]

and Thomas F. Krauss[a]

[a] School of Physics, Engineering and Technology, University of York, Heslington, York YO10 5DD, United Kingdom

[b] Procter and Gamble Technical Centres Ltd., Reading Technical Centre, Reading RG2 0QE, United Kingdom

[c] Department of Physics, Dhenkanal Autonomous College, Dhenkanal 759001, Odisha, India

Corresponding author's email address: Nyasha.Suliali@york.ac.uk

[*]These authors contributed equally to the work

Keywords: Optical sensor, guided mode resonance, bowtie grating, temperature compensation, mechanically robust sensor

---

1. Sensor fabrication and mechanical vibration analysis

1.1. Sensor materials, fabrication and simulation

Numerical methods were carried out with RCWA and FDTD simulation to determine the chirped GMR's period and filling factor. The period of chirp GMRs covers 9 nm ranging from 428 nm to 434 nm with a filling factor of 70%. Si_3N_4 film with a thickness of 150 ± 1.2 nm on Borosilicate wafers were bought from Silson Ltd. Ebaeeam resist ARP 6200.13 (Allresist GmbH) were spin-coated on the substrate. E-Beam lithography was employed to define patterns afterwards, then Reactive Ion Etching (RIE) was carried out to transfer the pattern from ARP 6200.13 to Si_3N_4 film with a mixture of gas 14.5 CHF3: 12.5 SF6. Finally, the resist was removed in 1165 remover (MICROPOSIT) then rinsed in acetone and IPA.

1.2. Vibration noise reduction using bowtie chirped GMR

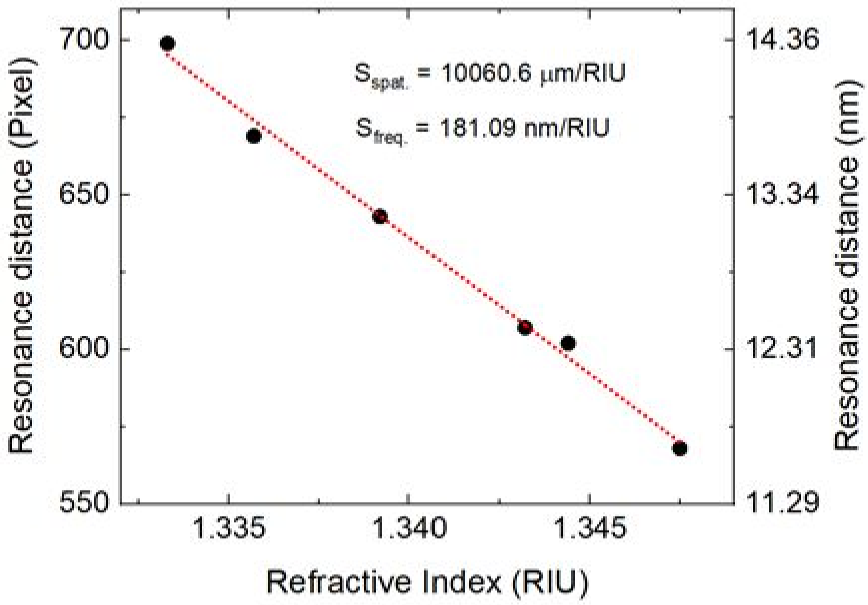

Figure S1: Sensitivity of bowtie chirped GMR sensor with different refractive indices corresponding to different concentrations of ethanol in water solution (0, 5, 10, 15, 20, 25% solutions). Note, the unit of frequency sensitivity is in nm, it is detected by spectrometer, the unit of the spatial sensitivity is in µm, it is obtained from the image captured by a camera.

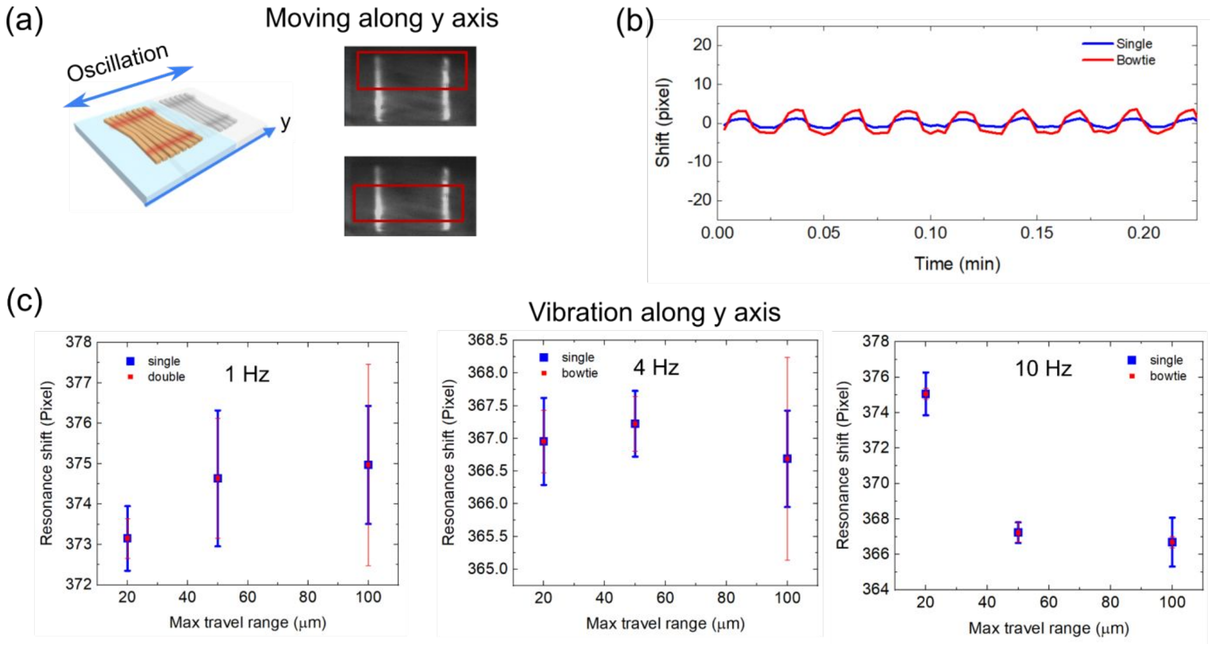

Figure S2: Mechanical vibration of the GMR sensor along the y axis. (a) The stage was vibrated along the y axis. The left image illustrates the movement direction for the bowtie chirped GMR sensor. The right image shows that the sample drifts away from the region of interest (ROI) which is indicated in the red rectangle. (b) The resonance position v.s. vibration time. The blue colour represents a single chirped GMR and the movement of the stage. The red colour represents the shift of the bowtie chirped GMR. The vibration frequencies are set to 40 Hz with a maximum range of 1 μm. (c) Different vibration frequency and maximum travel range are tested for the sensor. They are 1, 4 and 10 Hz respectively with 20, 50 and 100 μm maximum travel range.

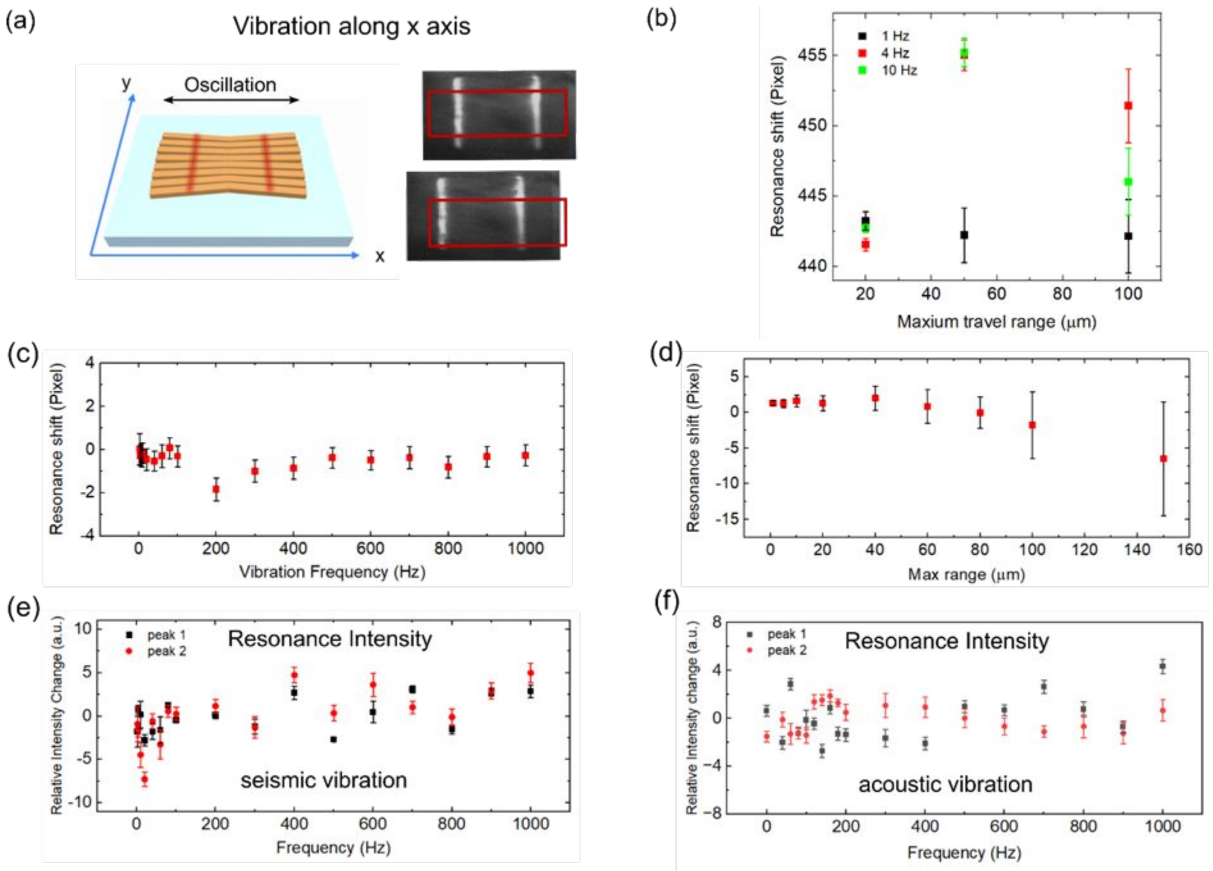

Figure S3: (a) Mechanical vibration along the x axis and an example of the resonance images (b) Vibration starts at three different frequencies and three different maximum travel ranges: 1, 4 and 10 Hz and 20, 50 and 100 μm respectively. The larger the maximum travel range is, the larger the error of the resonance shift. This is a combination of peak deformation and camera integration time. (c) The resonance shift of the bowtie chirped GMR against vibration frequency. The maximum travel range is set to 20 μm. (d) The resonance shift of the bowtie GMR against maximum travel range, from 1 μm to 150 μm (which is related to acceleration speed). The data were obtained from 300 full cycles in each measurement. (e) Dependence of relative resonance intensity on seismic vibration frequency. (f) Dependence of relative resonance intensity on acoustic vibration frequency. Note, the relative resonance intensity is calculated as the disparity between the actual resonance intensity and the average value for each dataset, this approach facilitates a clearer visualisation of error bars. A total of 25 repeat measurements were conducted at each frequency to ensure robust and comprehensive data collection.

We further performed the vibration test at different frequencies to see the stability of the bowtie configuration. As expected, the bowtie chirp GMR has stable performance (1 ± 0.5 px) across a large range of frequencies (1 - 1000 Hz) as shown in Fig. 2c. Especially for frequencies lower than 100 Hz, the resonance shift varies within half a pixel. Next, we investigated how varying the vibration acceleration speed impacts the stability of the bowtie GMR sensor. We employed different maximum travel ranges to represent corresponding acceleration speeds.

Figure 2d illustrates that the resonance shift increases notably with acceleration, particularly beyond a maximum travel range of 40 μm. This phenomenon can be attributed to the camera's integration time. With a fixed integration time (100 ms), a shorter sample travel distance results in the camera capturing a sharper image, thus enhancing the accuracy of the resonance position. In summary, the bowtie GMR performs optimally at lower vibration frequencies and reduced acceleration speeds.

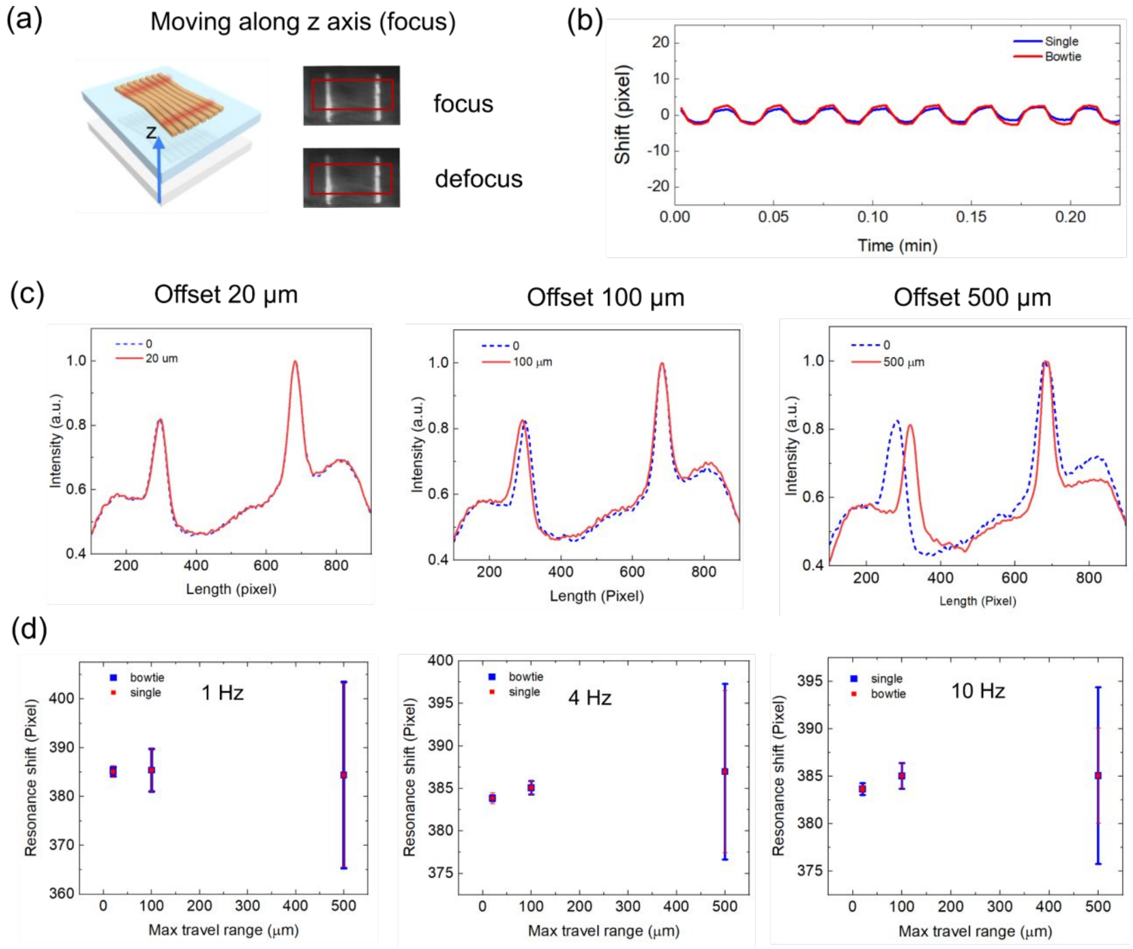

Figure S4: Vibration along the z axis. (a) Illustration of vibration direction and images at focus position or defocus position. (b) The resonance position v.s. vibration times. The blue colour represents a single chirped GMR and the movement of the stage. The red colour represents the shift of bowtie chirped GMR. The vibration frequencies are set to 40 Hz with a maximum range of 1 μm. (c) The resonance profiles at three different travel ranges 20, 100 and 500 μm. (d) The resonance shift at three vibration frequencies, 1, 4 and 10 Hz. The error bar presented the standard deviation of more than 300 full vibration cycles.

The defocus of the sensor causes the resonance profile to degrade; this effect is much more obvious when the sample is placed very far away from the focal plane. From Figure S4c, within the range of 20 μm offset at the focal plane, the bowtie chirp GMR shows less than 1 pixel

uncertainty. This larger uncertainty is caused by the resonance shape deformation, as can be seen in Figure S4c. When the offset is 500 μm, the resonance Full width at half Maximum is much broader than at zero offset.

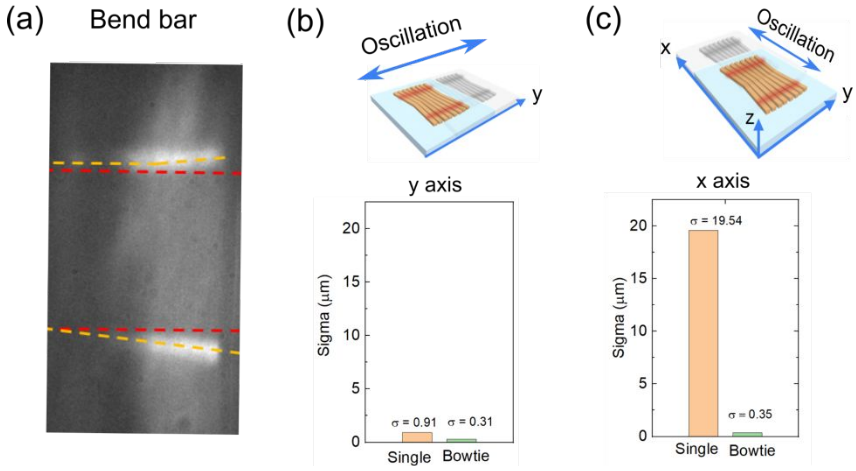

Figure S5: The comparison of the standard deviation of the resonance shift for bended bar chirp GMR (a). Top figure of (b, c) illustrates the vibration direction along the y and x axes respectively. Bottom figure of (b,c) shows the standard deviation of the resonance shift for single and bowtie chirp GMR when it is oscillating at 1 Hz with maximum travel range of 20 μm. It can be seen that bowtie chirp GMR can reduce the noise in both cases and especially has very low noise when vibration is along x axis.

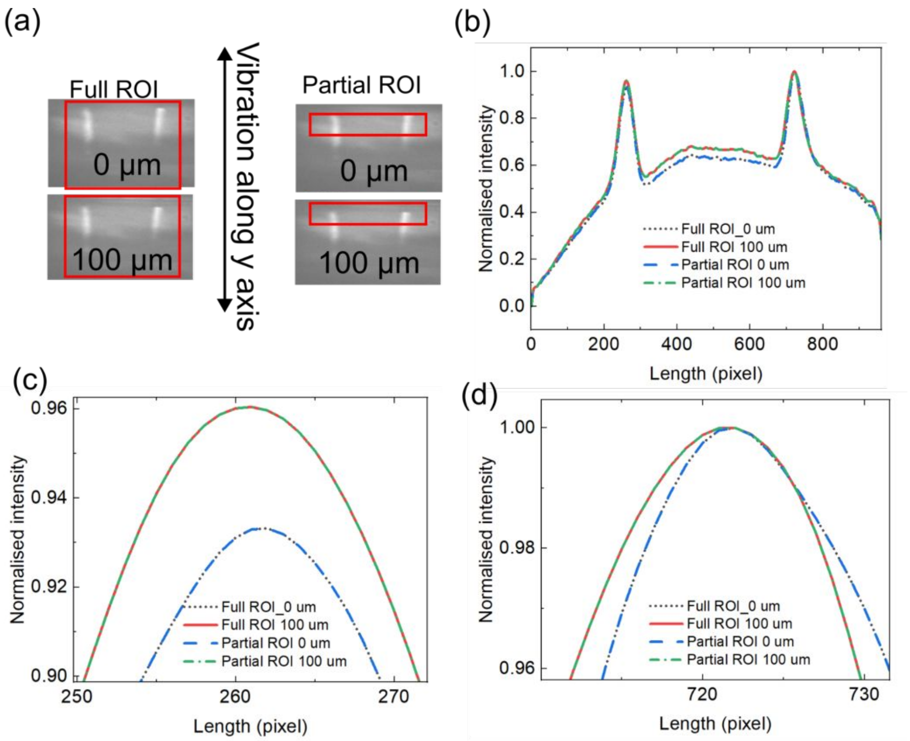

Figure S6: Effect of region of interest on the bended bar bowtie sensor. The sensor is vibrating along the y axis, (the direction of the bar), at 1 Hz with maximum travel range of 100 μm. (a) Illustration of the full region of interest v.s. partial region of interest. (b) the resonance profile obtained from the

corresponding ROIs overlaps each other, with only a slight difference in the intensity. (c) and (d) are zoomed in image of the left and right resonance from (b). The region of interest will not have a significant impact on the resonance shift of the bowtie chirped GMR design.

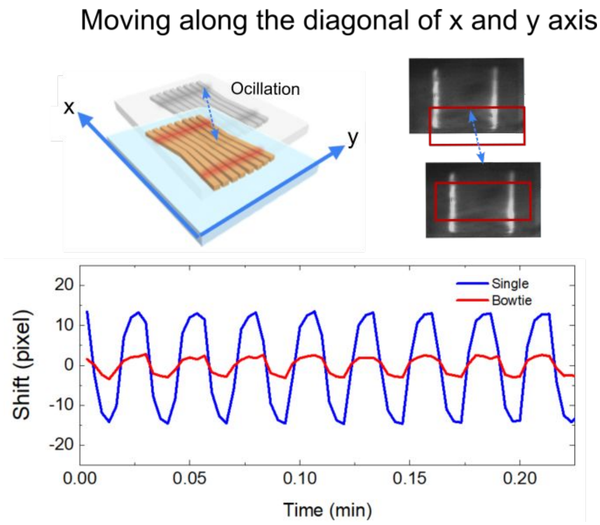

Figure S7: Vibration along the diagonal of the x and y axis. The top figure illustrates the direction of the movement, and the bottom figure shows the resonance position v.s. vibration times. The blue colour represents a single chirped GMR and the movement of the stage. The red colour represents the shift of bowtie chirped GMR. The vibration frequencies are set to 40 Hz with a maximum range of 1 μm. The vibration in the diagonal direction can be decomposed into vibrations along the x and y axis. Since the bowtie structure is good for reducing mechanical noise along the y axis, therefore, even if the vibration is in the diagonal direction, it can still be beneficial from the contribution of the y axis. For instance, compared with the single bar, it reduces the position error from 13.5 pixels to 2.8 pixels, and the standard deviation is reduced from 11.20 pixels to 2.17 pixels, reducing the error by 5 times.

1.3. Angle response for bowtie chirped GMR

Alignment errors often manifest themselves as angular misalignment, such as oblique incident. Here, we take two cases to illustrate its effect.

The grating equation for oblique incident light is

$$\frac{P}{m\lambda_0} = \frac{1}{\pm\sqrt{n_{eff}^2 - (\sin\theta\sin\phi)^2} - \sin\theta\cos\phi}$$

where (θ,ϕ) are angles of incidence in spherical coordinates, m is the diffraction order, n_{eff} is the effective refractive index, and P is the period.

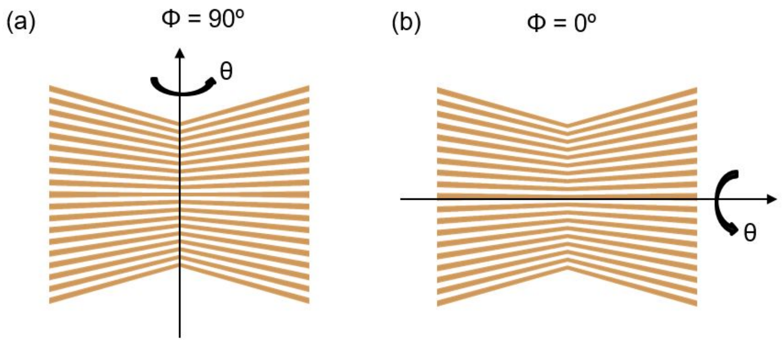

Fig S8: Illustration of rotation angle: (a) when Φ = 90°, the rotation axis is perpendicular to the grating strips. (b) when Φ = 0°, the rotation axis is parallel to the grating strips.

(1) When $\phi = 90^{\circ}$, as shown in figure S6(a) the grating equation becomes

$$\frac{P}{m\lambda_0} = \frac{1}{\pm\sqrt{n_{eff}^2 - \sin^2\theta}}$$

The change in variation for the period is an even function of θ. Thus, the displacement of the bars should go towards positive periods.

(2) When $\phi = 0^{\circ}$, as shown in figure S6(b), the grating equation becomes

$$\frac{P}{m\lambda_0} = \frac{1}{\pm(n_{eff} - \sin\theta)}$$

higher diffraction orders coupled into the ± 1 mode and form a bandgap[1], the resonance will stay in that band. The period's response will follow the same trend, both sides of the GMR will experience the same momentum/angle of incidence,.

Subsequently, we quantified the angular tolerance under a common condition, where the tilting axis isn't aligned with the rotation axis. We accomplished this by tilting the stage angle from 0 to 1 degree and recording the corresponding resonance positions (as depicted in Figure S8 e and f). The bowtie configuration exhibits an almost consistent resonance response even across the entire examined range, with a variation of only 5 ± 2.7 pixels. In contrast, the single grating demonstrates significant angular dependence, with a variation of 130 ± 41.2 pixels. Notably, for angles less than 0.6 degrees, the bowtie resonance exhibits a variation of less than 1 pixel.

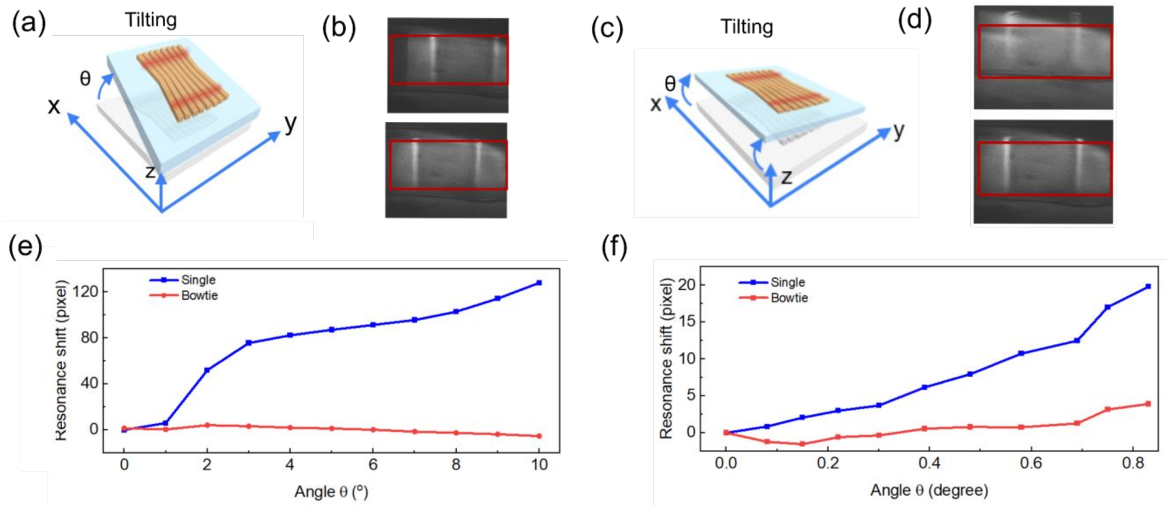

Figure S9: Angular tolerance test for bowtie chirped GMR. The GMR chip is placed on a 2 inch kinematic mount (Thorlabs). The geometry specification offers a maximum tilt angle of 1 degree. (a) and (c) represent a 3D sketch of the tilt angles along different axes, (b) and (d) represent the corresponding resonance images captured by the camera, (e) and (f) show the corresponding resonance shift. The blue curve represents the resonance shift on a single chirped GMR as a function of tilt angle. The red curve represents the shift of the bowtie chirped GMR. The maximum resonance position shift of single chirp GMR compared to the bowtie chirp GMR is 19.8 pixels and 3.9 pixels, with standard deviation of 6.67 pixels and 1.69 pixels. The bowtie chirped GMR can maintain a maximum position shift of less than 1 pixel within 0.7 degree. Overall, the bowtie chirped GMR reduces the noise fourfold.

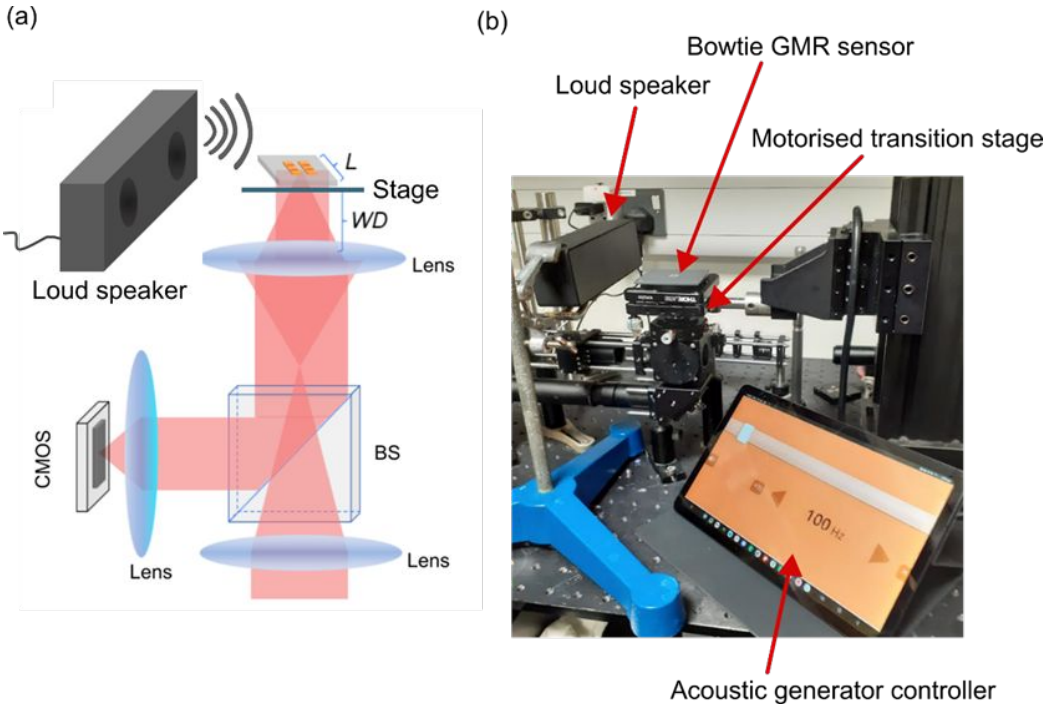

Figure S10: Acoustic vibration setup. (a) Sketch of the acoustic vibration setup. (b) photos of the acoustic vibration setup. The loud speaker can generate 103 dB random noise at various frequencies, and is placed 5 cm away from the sensor.

2. Temperature compensation

2.1. Fabrication of NOA1375 coatings and characterisation

To eliminate TOEs that would result in a shift in the resonance, the bowtie-chirped GMR sensor was partially coated. The coating was fabricated such that when illuminating the grating, the geometric position of the resonance on the chip is the same as that on the uncoated gratings which interact with the material under investigation.

Three of 6-gratings (in a 3×2 arrangement) were coated with Norland NOA1375 optical adhesive purchased from Edmund Optics. The as-fabricated sensor (Si_3N_4 gratings on borosilicate glass) was first cleaned in Piranha solution, a 3:1 ratio of 96% H_2SO_4 from Acros Organics to 35 wt% H_2O_2 from Thermo Scientific and sequentially rinsed in deionised (DI) water. The NOA1375 solution was brought to room temperature and 0.5 mL spin-coated onto the wafer at 500 rpm for 10 s and then 1000 rpm for 30 s using the Electronic Microsystems 4000 bench top spin-coater. Pre-exposure baking was done on a Stuart SD160 hotplate at 85 °C for 3 minutes. Photolithography was carried out on the Kloe Dilase 650 by exposing a rectangular region (covering 3 gratings) with a 375 nm source with a 10 µm spot. Post-exposure baking was performed at 90 °C for 2 minutes and the sample left to cool to room temperature. The un-cured parts of the coating were then removed in acetone for 1 minute, and immediately rinsed in DI water and dried with a gentle N_2 stream, leaving a cross-linked rectangular NOA1375 region.

Through this process, the thickness of the coating was designed to be 1 µm so as to completely encapsulate the evanescent tail of the guided mode beyond the 1/e point (180 nm as shown in the simulation on Fig. S2). In this configuration, the resonance shift on the coated gratings acts as the temperature reference signal, as the refractive index changes of the coating with which the evanescent tail solely interacts, are due to the TOE and not a change in analyte composition. An image of the sensor was obtained using a Leica DM4000 M LED materials microscope operated in the brightfield mode at a magnification of 5x. The thickness of the coating was measured by line profilometry using a Bruker Dektak profilometer with a stylus force of 5 mg and velocity of 10 µm/s.

2.2. Temperature compensation setup and data processing

For temperature sensing, Fig. S10 shows the experimental setup. In Fig. S10(a) the coated bowtie chirped GMR sensor (S) is glued onto the base of a flow beaker (B), using polymethyl methacrylate (PMMA) 950 resin and heating at 180 °C for two 10 s intervals to drive off the solvent from the PMMA.

Fig. S3(b) shows the temperature measurement setup. A heater powered by a Hilitand MH1210A temperature controller was connected to a reservoir (R) with 300 mL of 18.2 MΩ.cm deionised (DI) water, from which the solution was circulated through the beaker by a peristaltic pump (P). To measure the temperature of the solution as a function of time, a DFRobot DFR0198 waterproof digital temperature sensor was connected to an Arduino Uno R3 through a DFRobot DFR0055 adapter. The Arduino sensing and serial data generation code is provided by DFRobot[2]. The optical setup to obtain images of the guided mode resonance images was miniaturised and packaged into a 9 cm x 8 cm x 4 cm box[3]. A standard computer (PC) was used to control the Arduino, record temperature data, control the GMR box and process resonance images to obtain resonance data.

The DI water was heated from ~15 to ~30 °C for one hour and ten minutes and then the heater switched off for the sample to cool for a further 1.5 hours. Critical steps in data processing included computing the pixel (px) positions of peaks of the resonance intensity profiles using Python 3.11, matching the sampling rate of the resonance position data to that of the temperature measurements by interpolation in Origin Lab 2022 and calculation of the absolute shift from the double-bar data. Measurements obtained from the sensor are continuous time-varying signals which are converted to electrical signals in conventional instrumentation, hence the voltage symbol V will be used to refer to the resonance signals for brevity. The signals considered are the:

- measured TOE-dependent resonance shift on T ($V_T \propto \Delta n_T$),
- TOE-dependent resonance shift on M during calibration ($V_{CAL} \propto \Delta n_T$),
- measured composite resonance shift on M ($V_M \propto \Delta n_T + \Delta n_C$) and
- the calculated temperature compensated composition signal $V_C \propto \Delta n_C$.

Since the uncoated gratings are in a solution which refractive index also changes with temperature, calibration was carried out to correlate the TOE of water to that of the Norland coating. The correlation factor, hereafter referred to as the temperature compensation factor □ = V_{CAL}/V_T, was calculated. This step eliminates the need to measure temperature, thus reducing the sensing system to a 2-signal problem, V_T and V_M. If temperature fluctuates during measurements, Δn_T is cancelled by subtracting the temperature reference signal V_R = □V_T

(TOE on M) from the total/composite resonance shift of the uncoated sensor (V_M) to get the differential, composition dependent temperature compensated signal $V_C = V_M - V_R$. A simulation of the calculation using basic mathematical functions is shown in Fig. S4. This technique effectively decouples Δn_C from the measurements, making the guided mode resonance sensor thermally versatile.

Pertaining the direction of resonance shift, it is important to recall that the sensing mechanism of the chirped GMR chip is reduced from sensing a spectral shift for a given period, to sensing the position of the same spectrum coinciding with the matching period along the chirp. The direction of displacement therefore corresponds to the TOE as follows: When temperature rises, thermal expansion results in a drop in mass density, viscosity and consequently refractive index of the coating. Where refractive index drops, the supposed blue shift in resonance translates to the same wavelength resonating at a spatial position at a shorter period, which according to the design in Fig. 2 is towards the edges of the chip. The resonance shift due to increasing temperature is therefore opposite to that due to increasing concentration.

2.3. Bow-tie and temperature-compensated bacteria growth

Staphylococcus aureus were prepared using BioBall MultiShot 10E8 (Biomérieux), a water soluble ball containing between 0.7 and 1.5 x 10E8 *S.aureus*. The BioBall was added to 1 mL BioBall rehydration fluid (Biomérieux) and gently shaken until dissolved. Due to the unknown composition of the BioBall solution, it was important to remove all additional proteins, as these interfered with the sensor signal. To do so, the BioBall solution was centrifuged at 5000 RPM for 5 minutes using an Eppendorf Minispin centrifuge system, to form a pellet of bacteria. The supernatant was then removed and the pellet reconstituted in phosphate-buffered saline (PBS) solution. These steps were carried out three times to ensure all additional components of the BioBall were removed. Tryptic-Soy-Broth (TSB) was prepared at 30 g/L in MilliQ water using TSB powder and then the filter sterilised using a 0.2 micron syringe filter.

Bacteria growth measurements were carried out by affixing a sterile micro-well (Ibidi - 80366) on the sensor, injecting 30 µL PBS, 50 µL of the *S.aureus* BioBall solution, and 20 µL TSB (30 g/L) into the well and measuring the resonance shifts V_M and temperature resonance shifts V_T for 25 hours. The stage of the measurement setup was programmed to oscillate 100 um at a frequency of 0.2 Hz in the X axis to simulate mechanical movement. A heater was switched periodically throughout the experiment to simulate a varying temperature of the external environment. Fig. 6 in the main manuscript shows the measurement over 25 hours.

Other figures

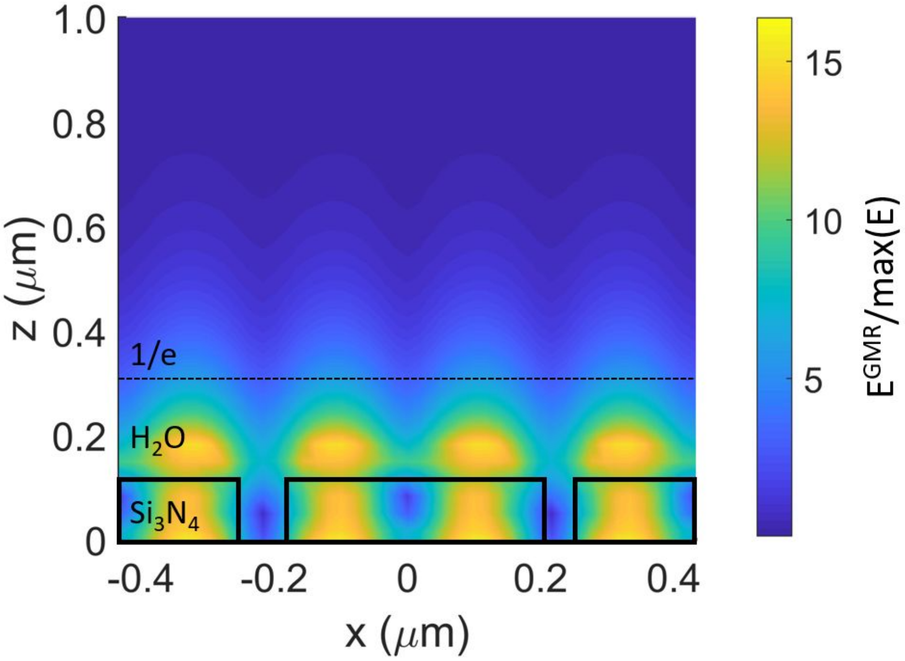

Figure S11: Cross section of the electric field distribution of a guided mode resonance. Simulated in Matlab for a 150 nm-thick Si_3N_4 grating with a period of 434 nm and 70% fill factor. The 1/e point of the intensity is 180 nm above the grating, shown in black dotted line.

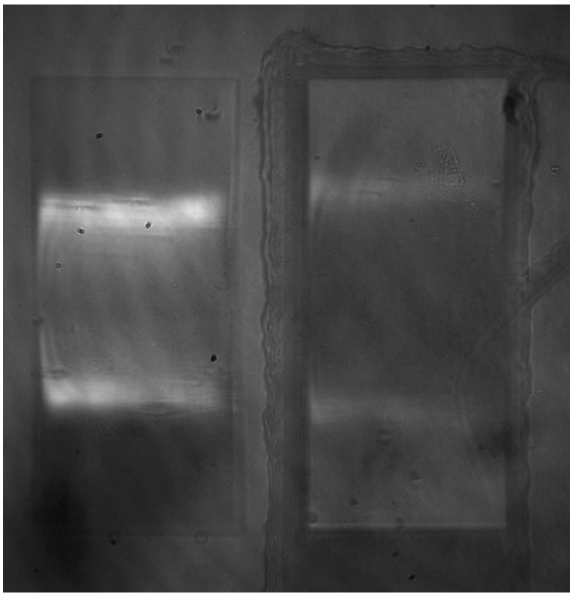

Figure S12: A typical resonance image of gratings T and M showing a typically low contrast resonance on T compared to M. The difference in intensities arises from the difference between the refractive index contrast of the grating-water and of the grating-coating interface.

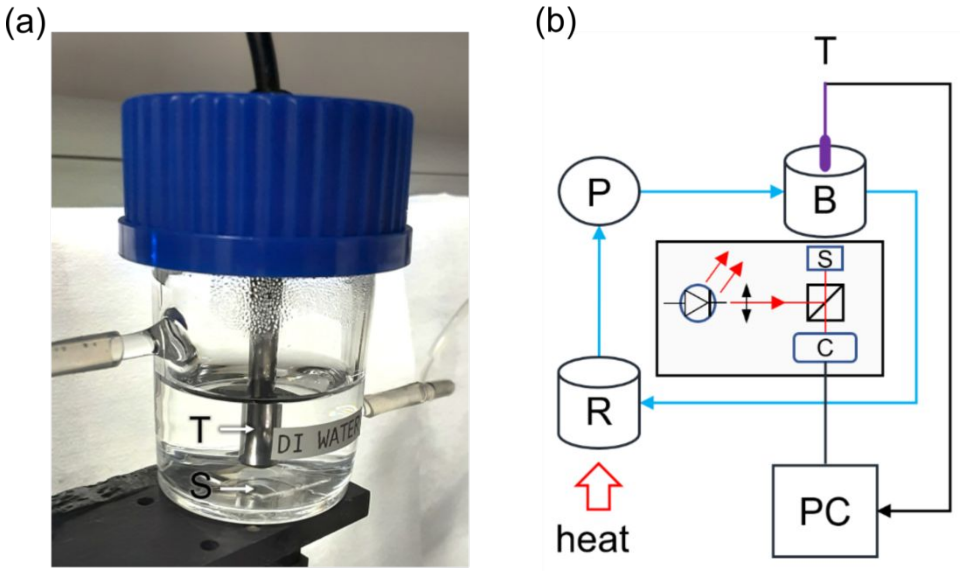

Figure S13: (a) A photograph showing the position of the GMR sensor S and temperature probe T in the flow beaker. **(b)** The temperature compensation setup where R is the reservoir, P-peristaltic pump, B-beaker, T-temperature probe, S-GMR sensor, C-camera, PC-computer. In the salinity measurement experiment, the sensor was glued onto a beaker and 300 mL of DI water circulated. Mechanical noise was introduced by vibrating the sample at 0.5 Hz along the grating throughout the experiment and thermal noise by heating the sample from 15 to 30 °C and then cooling it down to ~20 °C. The salinity of the solution was increased from 0 to 5 % (0 - 0.9 M NaCl) in 15 minute intervals for 2 hours. In this concentration range, the dn/dT (and consequently α) is the same as that of DI water from 0 to 90 °C[4]. To test a reverse change in composition, additional DI water was added twice in 15 minute intervals to reduce the salinity.

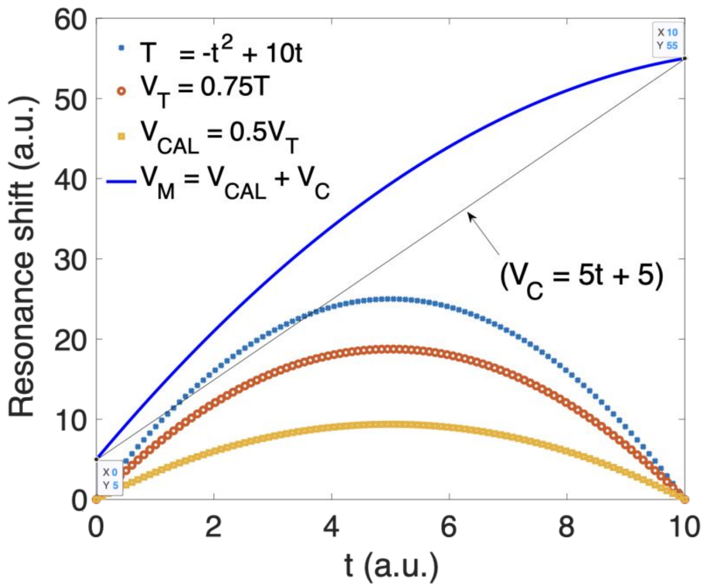

Figure S14: A simulation of the temperature compensation signal processing technique where the resonance shifts on grating M during calibration (V_{CAL}) and grating T (V_T) follow the temperature trend T. The calibration signal V_{CAL} is a fraction of V_T, the arbitrary fraction here (0.5) corresponds to the ratio

of TOE coefficients. Assuming that the composition or concentration of the analyte increases linearly as shown by the signal V_C, the experimentally measured signal V_M is therefore the sum V_{CAL} + V_C. Therefore, the objective of the signal processing stage is to find V_C after measurements of V_M and V_T are obtained.

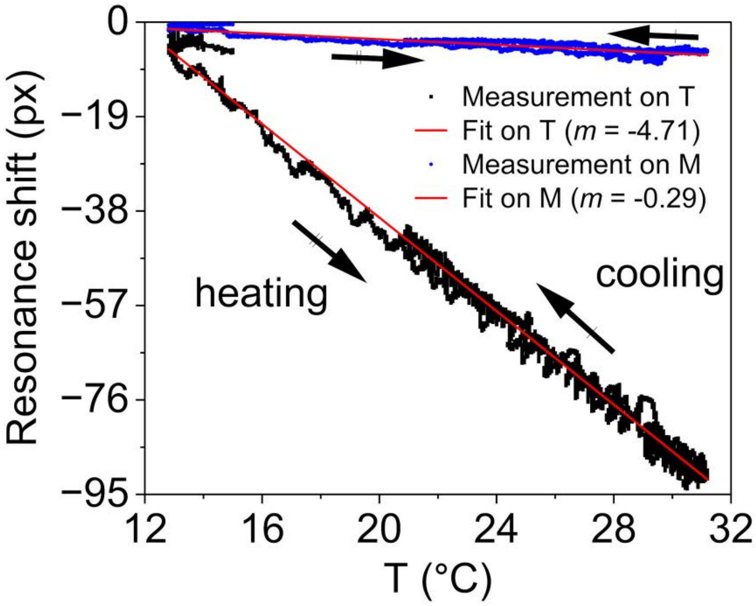

Figure S15: The resonance shift plotted as a function of temperature is linear. The data points trace the resonance shifts during the heating and cooling cycles, while a line of fit provides regression data from which sensitivity and the magnitude of the TOE coefficients can be determined. The negative slope indicates reduction of n_T with temperature. The rate of the resonance shift can therefore be obtained by either calculating a ratio of amplitudes of resonances to temperature in Fig 4(b) (4.89 px/°C for T) or the slope *m* of the graph i.e. *m* = - 4.71 px/°C with R^2 = 98.98%. On the other end, the resonance shifts on M result in a temperature sensitivity of 0.29 px/°C. After determining the refractive index sensitivity of the sensor through the salinity measurement experiment, the TOE coefficients were determined.

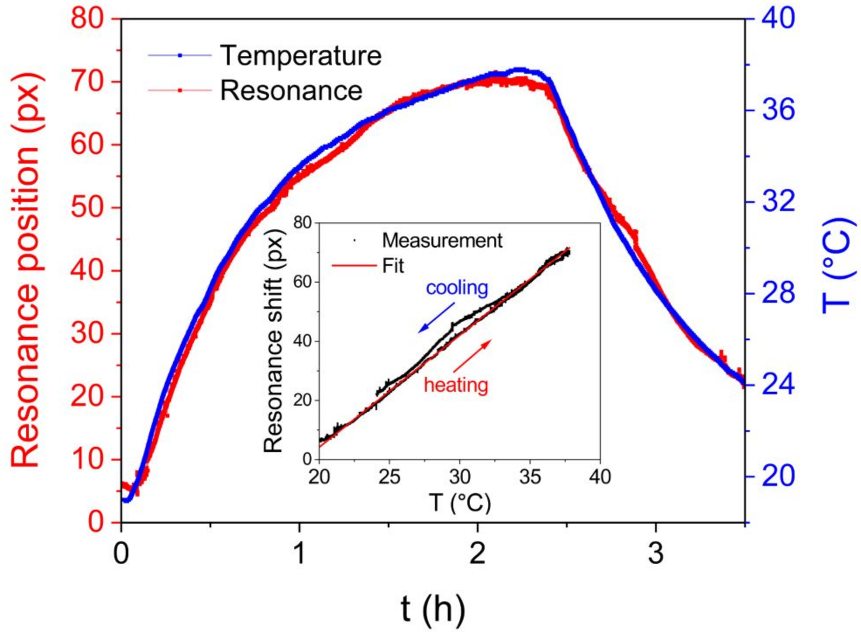

Figure S16: Resonance response to temperature variation where temperature fluctuation during heating/cooling is minimal over a long period.

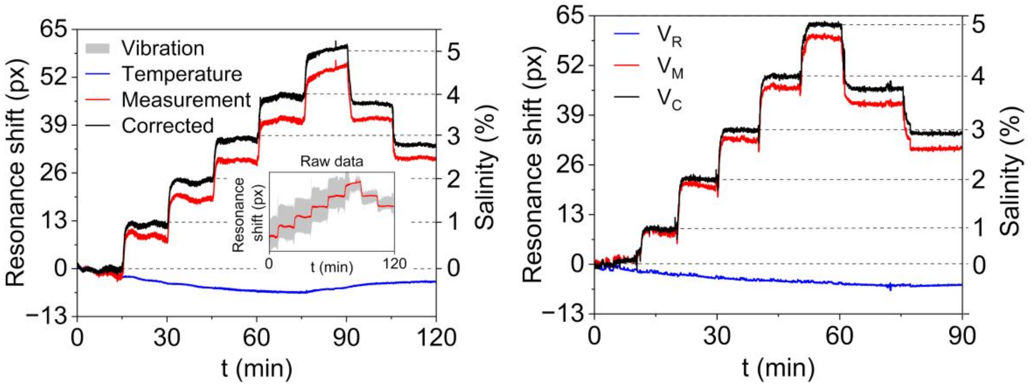

Figure S17: Resonance measurements of V_C obtained at 10 minute intervals in the first hour and then at 15 minute intervals while temperature is raised from 15 to 30 °C without vibrations. The measurements are in agreement with those obtained as the sensor vibrated at 0.5 Hz (see video clip in supplementary media). The results show step changes in the composition as expected (when chemical equilibrium is reached within 180 nm of the surface of the sensor surface). The inset shows the shaded raw measurements due to mechanical vibrations, which heavily modulate V_M making it very noisy. After simple subtraction, the noise is reduced by 97.3% to get the corrected V_M. Temperature also modulates the measurement. In the range 15 < t < 30 min for instance, the graph of V_M shows a decrease in V_M from 13 px when the saline solution enters the beaker, as though n_C is decreasing. The reversal is clearly a thermal effect, which is corrected to get V_C. The temperature reference signal V_R increases (heating) in the negative because dn_T/dT for water is negative[4–9] i.e. the resonance shifts towards lower period regions of the chirped gratings. The gap between V_C and V_M clearly follows the temperature

trend as it increases up to the 75th minute and tapers off during cooling. For comparison, control measurements obtained without mechanical vibrations are shown in Fig. S7 are in agreement with measurements in Fig 4(d). To correlate resonance shift measurements to the refractive index unit (RIU), the refractive index of the saline solution was measured using a Reichert Brix-RI-Check refractometer. The results presented in Fig. S8 were used to calculate the TOE coefficient of water and the NOA1375 coating which are -4.3 x 10^{-5} RIU/°C and -9.4 x 10^{-3} RIU/°C respectively. The calculated TOE coefficient is approximately half of the value previously obtained at 633 nm (-8 x 10^{-5} RIU/°C)[8,10] and comparable to the constant term obtained at 1550 nm (-4.1 x 10^{-5} RIU/°C)[11].

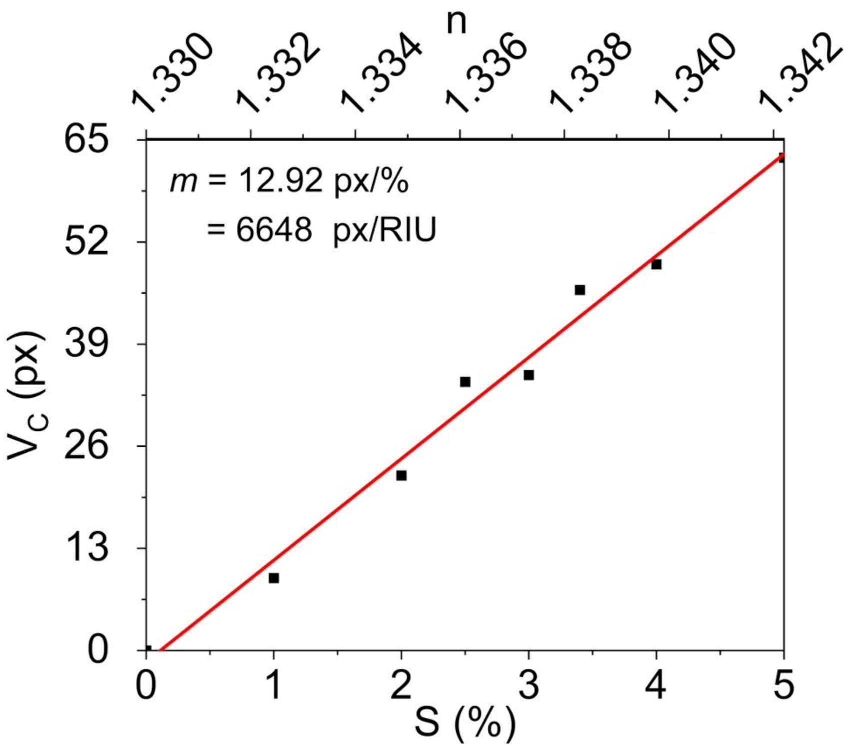

Figure S18: Resonance measurements of temperature compensated resonance on M (V_C) correlated to the RIU to verify sensitivity of the sensor. Two important parameters can be deduced from this salinity experiment: the TOE coefficient of water (dn_M/dT) from the sensitivity measurements of grating M (0.29 px/°C) and that of NOA1375 (dn_T/dT). The value of dn_M/dT confirms the veracity of measurements because it has been reported before, while dn_T/dT is a new benchmark parameter to use to determine dn/dT for any given analyte in contact with grating M. To verify the RIU sensitivity of the sensor, measurements V_C against n (RIU) are presented in Fig. S8. The data are clearly linear, such that the sensitivity can be deduced from the slope *m* = 6648 px/RIU, giving dn_M/dT = -4.3 x 10^{-5} RIU/°C at our illumination wavelength of 647 nm. The calculated TOE coefficient is approximately half of the value obtained at 633 nm (-8 x 10^{-5} RIU/°C)[8,10] and comparable to the constant term obtained at 1550 nm (-4.1 x 10^{-5} RIU/°C)[11]. The value of dn_T/dT can be used to determine dn/dT for other analytes through a procedure similar to the calibration experiment described in section 2.3 (composition is constant during calibration). Since $\alpha = V_{CAL}/V_T$ and pixel shifts can be converted to RIU through the sensitivity *m* = 6648 px/RIU (see Fig. S8), α is thus equally a ratio of the dn/dT of an unknown material to dn_T/dT = -9.4 x

10^{-3} RIU/°C from our measurements. In the last section of this report, measurements of bacterial growth in an environment with both mechanical and thermal noise are presented.

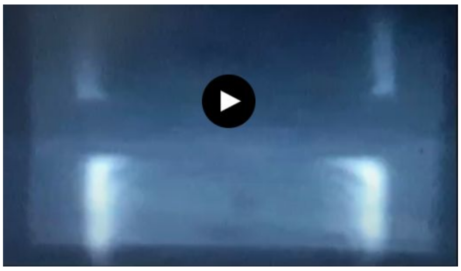

Sensor vibration video: Video clip showing vibration of the sensor along the x-axis during temperature compensated salinity sensing.

References

1. Novotny, L. Strong coupling, energy splitting, and level crossings: A classical perspective. *Am. J. Phys.* **78**, 1199–1202 (2010).
2. Waterproof_DS18B20_Digital_Temperature_Sensor__SKU_DFR0198_-DFRobot. https://wiki.dfrobot.com/Waterproof_DS18B20_Digital_Temperature_Sensor__SKU_DFR0198_.
3. Drayton, A. Autonomous photonic biosensor. (University of York, 2021).
4. Aly, K. M. & Esmail, E. Refractive index of salt water: effect of temperature. *Opt. Mater.* **2**, 195–199 (1993).
5. Kim, C.-B. & Su, C. B. Measurement of the refractive index of liquids at 1.3 and 1.5 micron using a fibre optic Fresnel ratio meter. *Meas. Sci. Technol.* **15**, 1683 (2004).
6. Zhang, Y., Xue, J., Liu, W., Zhang, Y., Liu, Z., Lai, B., Zhang, J., Yang, X. & Yuan, L. Measurement of liquid thermo-optical coefficient based on all-fiber hybrid FPI-SPR sensor. *Sens. Actuators Phys.* **331**, 112954 (2021).
7. Novais, S., Ferreira, M. S. & Pinto, J. L. Determination of thermo-optic coefficient of ethanol-water mixtures with optical fiber tip sensor. *Opt. Fiber Technol.* **45**, 276–279 (2018).
8. Kamikawachi, R. C., Abe, I., Paterno, A. S., Kalinowski, H. J., Muller, M., Pinto, J. L. & Fabris, J. L. Determination of thermo-optic coefficient in liquids with fiber Bragg grating refractometer. *Opt. Commun.* **281**, 621–625 (2008).
9. Yang, Y., Zhao, Q., Guo, Z., Wu, X., Cheng, K., Chen, Z., Jiao, J. & Tu, X. Phase Transition Monitoring of Liquid Metal Based on Temperature Sensors of Packaged Microbubble Resonators. *IEEE Sens. J.* **23**, 8178–8183 (2023).
10. Solimini, D. Loss Measurement of Organic Materials at 6328 Å. *J. Appl. Phys.* **37**, 3314–3315 (1966).

11. Kim, Y. H., Park, S. J., Jeon, S.-W., Ju, S., Park, C.-S., Han, W.-T. & Lee, B. H. Thermo-optic coefficient measurement of liquids based on simultaneous temperature and refractive index sensing capability of a two-mode fiber interferometric probe. *Opt. Express* **20**, 23744–23754 (2012).
